# Xanthomes historiques révélant une hypercholestérolémie familiale de type IIa

**DOI:** 10.11604/pamj.2015.21.252.6631

**Published:** 2015-08-06

**Authors:** Fayçal El Guendouz, Hicham Baïzri

**Affiliations:** 1 Service d'Endocrinologie Diabétologie et Maladies Métaboliques, Hôpital Militaire Avicenne, Marrakech, Maroc

**Keywords:** Xanthomes, coude, Hypercholestérolémie, xanthomata, elbow, Historical xanthomata revealing a familial hypercholesterolemia type IIa

## Image en medicine

Un garçon de 12ans, issu d'un mariage consanguin au premier degré. Il rapporte l'apparition à l’âge de 5 ans de formations sous-cutanées, indolores, au niveau des coudes, des genoux, des fesses et des pieds qui augmentaient progressivement de taille. L'examen cutané a objectivé de multiples xanthomes, tubéreux et tendineux, fermes et indolores siégeant en regard des coudes (A), des genoux (B), des fesses (C), des tendons d'Achille (D) et des articulations inter-phalangiennes des pieds (E). La plainte essentielle du patient était esthétique. L'examen ophtalmologique n'a pas trouvé d'arc cornéen ou de gérontoxon. Le bilan lipidiquea confirmé le diagnostic d'une hypercholestérolémie majeure type IIb avec un taux de cholestérol total à 7,10 g/l, un taux de LDL à 6,50 g/l, un taux de HDL à 0,42 g/l et un taux de triglycérides à 1,13 g/l. Le bilan de retentissement cardiaque n'a pas trouvé de dépôts valvulaire à l’échographie cardiaque, ni des signes de souffrance coronaire à l'preuve d'effort. L'exploration des troncs supra-aortiques, des membres inférieurs et des artères rénales ne montre pas d'anomalies. Le patient a bénéficié en plus d'une diététique à visée hypolipémiante, d'un traitement à base d'atorvastatine associé à l’ézétimibe et l'aspirine à la dose de 75mg/j. Nous avons noté une nette amélioration du bilan lipidique: Cholestérol total à 3,40 g/l et LDL à 2,92 g/l. Le patient a bénéficié aussi d'une cure chirurgicale des xanthomes les plus disgracieux et gênants. Aucun retentissement cardiovasculaire n'a été noté durant l’évolution et le taux de LDL fluctuait aux alentours de 3 g/l sur une période 4 ans.

**Figure 1:**
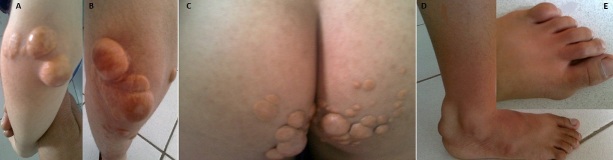
A= Xanthome tubéreux du coude; B= Xanthome tubéreux du genou, C= Xanthome tubéreux des fesses; D= xanthomes tendineux; E= inter-phalangiennes des pieds

